# Electric Vehicle Charging Route Planning for Shortest Travel Time Based on Improved Ant Colony Optimization

**DOI:** 10.3390/s25010176

**Published:** 2024-12-31

**Authors:** Aiping Tan, Chang Wang, Yan Wang, Chenglong Dong

**Affiliations:** School of Cyber Science and Engineering, Liaoning University, Shenyang 110036, China; aipingtan@lnu.edu.cn (A.T.); 4032232409@smail.lnu.edu.cn (C.W.); 4032232392@smail.lnu.edu.cn (C.D.)

**Keywords:** electric vehicles, route planning, ant colony optimization

## Abstract

Electric vehicles (EVs) are gaining significant attention as an environmentally friendly transportation solution. However, limitations in battery technology continue to restrict EV range and charging speed, resulting in range anxiety, which hampers widespread adoption. While there has been increasing research on EV route optimization, personalized path planning that caters to individual user needs remains underexplored. To bridge this gap, we propose the electric vehicle charging route planning based on user requirements (EVCRP-UR) problem, which aims to integrate user preferences and multiple constraints. Our approach utilizes topology optimization to reduce computational complexity and improve path planning efficiency. Furthermore, we introduce an improved ant colony optimization (IACO) algorithm incorporating novel heuristic functions and refined probability distribution models to select optimal paths and charging stations. To further enhance charging strategies, we develop a discrete electricity dynamic programming (DE-DP) algorithm to determine charging times at efficiently chosen stations. By combining these methods, the proposed IACO algorithm leverages the strengths of each approach, overcoming their individual limitations and delivering superior performance in EV routing and charging optimization.

## 1. Introduction

As a clean and sustainable mode of transportation, new energy vehicles have attracted widespread attention and research worldwide [1,2]. With the increasing severity of climate change, air pollution, and energy security issues, new energy vehicles have emerged as a critical solution to reduce greenhouse gas emissions, improve air quality, and reduce reliance on finite fossil fuels [3]. Promoting carbon neutrality has become a significant initiative for many countries. Electric vehicles (EVs) represent the primary direction of new energy vehicle development. According to [4,5], several countries have announced plans to phase out internal combustion engine vehicles in the near future. For example, Norway has stated that all cars and vans sold by 2025 should be zero-emission. India, Israel, and the Netherlands have announced that all cars sold by 2030 will be electric, while Germany and the UK have extended their targets to 2040. Taiwan, the United States, and China aim for 2040 or earlier. These initiatives will have a profound and far-reaching impact on the energy, environmental, and automotive sectors. EVs are projected to account for 58% of new car sales by 2040 [6]. Therefore, research related to EVs is of great importance. However, the EV market still faces several challenges [7,8,9], including insufficient charging infrastructure, limited battery life, and constraints in battery technology. The resulting range anxiety and charging difficulties have negatively impacted user experience and hindered the growth of the EV market.

To address the limited cruising range of electric vehicles (EVs), various types of charging infrastructure have been developed [10]. In addition to large-scale fast charging stations [11], household chargers and parking lot charging piles have become essential for recharging EVs [12]. Beyond hardware, software applications based on the Internet of Vehicles (IoV) [13] provide EV owners with services such as high-precision electronic maps, charging station locations, and accurate route navigation [14,15]. However, these navigation systems often fail to address the range anxiety experienced by EV drivers.

Current research on electric vehicle path planning primarily addresses the electric vehicle routing problem (EVRP) [16] and optimization of charging infrastructure [17]. However, these studies often focus on the global optimization of paths for multiple vehicles or the layout planning of charging stations. Less attention has been paid to the charging path planning of a single electric vehicle, especially regarding personalized user needs, which remains underexplored. In contrast, our work aims to optimize the driving path of an individual electric vehicle from the user’s perspective, considering factors such as charging station locations, charging power, the vehicle’s remaining battery life, queuing time estimation, in-car energy consumption, and user-reserved electricity. The goal is to provide an optimal path solution that balances charging requirements with travel efficiency. This approach better aligns with actual user behavior and significantly enhances the user experience of electric vehicle usage.

This paper proposes an electric vehicle charging route planning problem based on user requirements (EVCRP-UR), aiming to comprehensively consider user needs and multiple constraints to provide users with a route with the best travel time. Unlike existing studies, our problem model is more comprehensive and introduces innovations in the following aspects. First, we consider the queuing problem [18] at charging stations and replace the traditional queuing model with appointment charging to accurately calculate the queuing time to reach the charging station. In this way, we avoid the results being affected by the queuing time estimation error and improve the user experience. Second, we consider whether or not the user turns on the in-car electrical appliances (such as air conditioning, navigation, etc.), incorporate the resulting energy consumption of electrical appliances into the model, and consider the impact of this on the overall power consumption. Finally, we allow users to flexibly set the reserved power requirements at the destination to meet the driving preferences of different users. By combining these constraints, the problem model proposed in this paper is more in line with the actual application scenario and provides theoretical support for personalized route planning.

In light of these challenges, the proposed EVCRP-UR problem becomes a complex one. It not only involves optimizing the driving route while satisfying constraints, but also considers the selection of charging stations and charging times. While it is possible to solve small instances of this problem using exact algorithms, such exact methods significantly increase computational time, making heuristic and metaheuristic methods more suitable [19]. However, no single algorithm can solve all problems. Although each algorithm has its advantages, they also have some disadvantages. Ant colony optimization [20] can handle graph-based pathfinding problems more effectively compared to other heuristic algorithms; however, it has inherent limitations, especially in queuing problems, charging time selection, and user preferences. Dynamic programming [21] can help us to choose charging time, but cannot help us plan the path.

For the reasons mentioned above, we address these shortcomings using a mix of different techniques. We propose a topology optimization method that can reduce the computational complexity of path planning and improve its efficiency. We improve upon the ant colony optimization algorithm to help choose appropriate paths and charging stations through new heuristic functions and probability distribution calculation methods. For each path, we design a discrete electricity dynamic programming (DE-DP) algorithm to plan the charging time of the charging stations reasonably. By combining the strengths of various optimization algorithms, we propose an improved ant colony optimization (IACO) algorithm that integrates the above algorithms to overcome their weaknesses and improve overall performance.

Summarizing, the main contributions of the paper are as follows:

- We propose an EV charging route planning problem focused on user needs and experience, which we call EVRCP-UR. The proposed model accounts for precise queuing time estimation using appointment charging, energy consumption of in-car appliances, and user-defined reserved power at the destination, providing personalized and practical solutions.
- We develop an improved ant colony optimization (IACO) algorithm with innovations including topology optimization, enhanced heuristic factors (e.g., charging station importance), a discrete electricity dynamic programming (DE-DP) algorithm for charging time planning, and a refined probability calculation for end-point handling.
- We validate our approach using Beijing’s road network and charging station data. The results show significant improvements over baseline methods, reducing travel time and enhancing user satisfaction.

The rest of the paper is organized as follows: Section 2 provides a brief overview of related work; Section 3 reports the methodological approach we adopted; Section 4 presents the exploited dataset, including data from the Beijing road network and public charging stations; Section 5 examines the findings; and the conclusions are presented in Section 6.

## 2. Related Works

Despite the growing popularity of EVs, many unresolved challenges remain. Most notably, EVs’ driving range is relatively short [22], currently constrained by limitations in battery technology [23], which ultimately hinders their convenience and reliability. Additionally, the number of existing charging stations is insufficient [24] and their distribution is uneven. Due to the uncertainty of charging time, location, and power demand, the stochastic nature of EV charging can lead to queuing issues and compromise the reliability of the power system [25].

To solve the above problems, researchers have studied how to solve the range anxiety problem of electric vehicle travel. Several papers have discussed battery swap technology. Adu-Gyamfi et al. [26] investigated Chinese residents’ adoption intention for battery swap technology for electric vehicles. Adu-Gyamfi et al. [27] explored consumers’ adoption intention for battery-swappable electric vehicles (BSEVs) and plans to use BSEVs for sustainable mobility. These studies analyzed the economic and environmental benefits of battery swap technology. However, they primarily focused on consumer perceptions and market potential, without delving into the operational and path-planning challenges associated with battery swap technology.

Meanwhile, other papers have discussed load optimization strategies for power networks under V2G. Zhang et al. [28] solved the problems of Internet of Electric Vehicles allocation and scheduling with joint vehicle-to-grid regulation service and edge computing service to flatten the power fluctuations of the city power grid. Zhang et al. [29] presented a multi-EV charging scheduling strategy based on charging station load balancing. This strategy effectively balances the load levels of various charging stations while reducing the overall system travel time. However, these studies only considered the operation of the power network.

A number of papers have discussed path-planning strategies from the perspective of charging stations. Luo et al. [30] proposed a more practical scheduling strategy for different types of EVs considering the convenience of drivers, road traffic speed, number of vehicles at the charging station, and charging network load. This plan can simultaneously reduce the wait time of charging and increase the operational efficiency of charging stations. Hou et al. [31] proposed an integrated framework for multiobjective EV path planning with varied charging pricing strategies considering the driving distance, total time consumption, energy consumption, charging fee, and other such factors. Their framework was designed from the perspective of charging stations to maximize the total revenue of charging stations and balance their respective profits.

In addition, path-planning strategies can take a multi-vehicle perspective in order to reduce the total charging and waiting times. Zhang et al. [32] formalized the scheduling problem of EV charging as a Markov decision process and proposed deep reinforcement learning algorithms to address it. The objective of the proposed algorithms was to minimize the total charging time of EVs and maximize the reduction in the origin–destination distance. Jha et al. [33] proposed a smart decision-hunting optimization (SDHO) algorithm for determining the optimal charging station for each EV. Kumar et al. [34] allocated a charging station to each EV by considering travel needs and battery specifics, with the objective of minimizing travel time, queue time, recharging time, and energy costs. An et al. [35] considered the problem of assigning each electric vehicle in a region to a charging station to minimize the total charging time. Their paper presented the earliest finish charging (EFC) algorithm to solve the charging path problem. They described the characteristics of electric vehicle charging behavior through three processes: from the origin to the charging station, while charging at the station, and from the charging station to the destination. The above method assigns a reasonable charging station to each vehicle in order to reduce the charging time of all vehicles. However, the authors only considered the situation in which each vehicle is charged only once, and did not consider the subsequent situation after charging, nor does their proposed approach conduct planning according to the overall itinerary of each vehicle. In addition, assigning charging stations to multiple vehicles means that route planning cannot be based on the needs of a single user.

The problem of charging route planning from the perspective of charging stations or multiple vehicles is actually a scheduling problem for electric vehicles; instead of actually planning the total journey of each vehicle, a charging station is assigned to each vehicle. Route planning problems are typically solved by applying shortest-path algorithms. Traditional route planning methods often rely on exact algorithms such as the Dijkstra algorithm [36] or Bellman–Ford algorithm [37], which aim to determine the shortest or fastest path between two points based on predefined metrics such as the distance or travel time. These algorithms operate under the assumption of static conditions, overlooking dynamic factors such as traffic congestion or user-specific constraints. While these methods are efficient for basic routing, they struggle to address complex multi-faceted problems that involve additional constraints such as energy consumption or charging station waiting time for electric vehicles. In such cases, heuristic algorithms have gained attention, including the A* algorithm [38], ant colony optimization [20], and genetic algorithms [39]. These methods offer greater flexibility by searching for near-optimal solutions in complex environments, thereby balancing computational efficiency with adaptability to diverse requirements. There are a number of current studies on path planning for single electric vehicles that focus on improving the traditional algorithms mentioned above. Perger and Auer [40] focused on energy-saving path planning for electric vehicles using the Yen algorithm [41], an improvement of the Bellman–Ford algorithm. They considered topography and battery lifetime to find an optimal solution. This method optimizes energy consumption and travel time, hoping to reach the destination with the highest possible state of charge; however, it does not take into account situations where charging is needed midway. Ding et al. [42] proposed a charging warning and path planning method for EVs with insufficient energy based on the Dijkstra algorithm. This method only considers finding the current optimal charging station when the battery is low. However, if multiple charging is required, selecting the local optimum each time may not result in the global optimum. Mansfield et al. [43] proposed a new detour and charging station selection scheme based on the A* algorithm and simple additive weighting (SAW) [44] to optimize EV routing during unexpected road events, achieving high success rates in simulations. However, the SAW-based charging station selection method lacks consideration of the overall trip, potentially leading to suboptimal decisions that fail to minimize total travel time and charging efficiency. Qi [45] proposed an optimization method for electric vehicle charging scheduling based on the ant colony optimization algorithm with adaptive dynamic search (ADS-ACO). The optimization goal of their study was to ensure that when users arrive at the charging station from their departure point, the remaining battery power of the user can reach the shortest time required for the entire road section of the charging station. However, focusing solely on minimizing the time to reach the charging station without considering the overall trip duration and multiple charging needs may lead to suboptimal charging strategies for longer journeys.

Although the above-mentioned studies have proposed path-planning methods for different problems, there have been few studies on path-planning for single electric vehicles from the user’s perspective. Moreover, the selection of charging stations mainly considers the current optimal situation (considering charging only once), and does not consider the possibility of multiple charging sessions to reduce travel time or situations where one charge is insufficient to reach the destination. Thus, these approaches are not flexible enough to select charging stations and charging time.

## 3. Methodology

### 3.1. Description of Problem

Unlike existing studies, we propose an electric vehicle charging route planning problem based on user requirements (EVCRP-UR), aiming to comprehensively consider user needs and multiple constraints to provide users with the optimal travel time route. Our proposal includes the following innovative contents:

- **Flexible reserved electricity requirements:** One significant innovation is the ability for users to set personalized reserved electricity requirements for their destination. Different users may have different preferences regarding the amount of remaining energy upon reaching their destination. For example, when the user arrives at home, there is a charging station available; thus, they may not want to charge too much before returning home. Alternatively, when the user arrives at a place where there is no charging station nearby, they may want to reserve more power. Our model allows for flexibly setting thre reserved power at the destination, offering a more tailored and personalized experience. This flexibility accommodates diverse user preferences and ensures that the planning process is adaptable to individual needs.
- **Energy consumption of electrical appliances:** Unlike many existing models that overlook the impact of in-car electrical appliances (air conditioning, navigation, etc.) on energy consumption, our model includes these factors as key elements. We take into account the energy consumption of these appliances, which can significantly affect the overall energy usage, and consequently the travel efficiency. This innovation ensures that charging route planning is more realistic and better aligned with actual user behavior, as the use of in-car appliances varies based on user preferences and environmental conditions.
- **Queuing problems:** Traditional studies often rely on simplistic queuing models or ignore the queuing time altogether, which can lead to inaccuracies in route planning. In contrast, our model replaces the traditional queuing approach with an appointment-based charging system. This allows us to accurately calculate the waiting time before reaching a charging station, thereby mitigating the impact of queuing time estimation errors. By incorporating real-time queuing information, we improve the accuracy of the charging route planning and enhance the user experience.

By combining these innovations, our proposed model is more aligned with real-world applications and provides theoretical support for personalized route planning.

In this subsection, we introduce the notation and definitions of the proposed EVCRP-UR problem. Table 1 provides a summary of some of the notation that we use in the problem definition. The detailed definition of the problem is laid out below.

**Definition** **1.** *Road network.*

**Figure 1:**
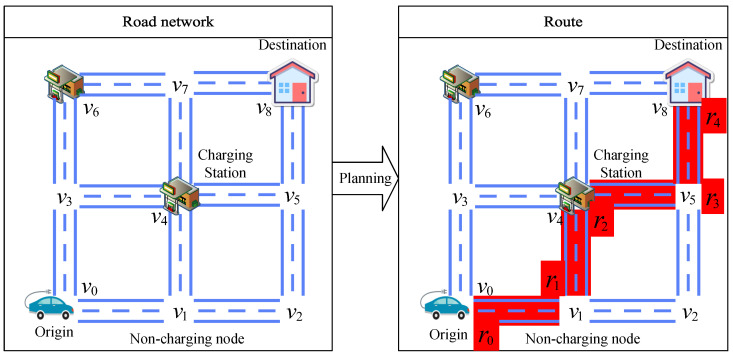
Road network and route diagram.

As shown in Figure 1, the road network is a graph G=〈V,E〉 consisting of nodes and edges; $V=\left\{v_{1},v_{2},\cdots ,v_{n}\right\}$ denotes the set of non-charging station nodes and charging station nodes, *n* is the number of nodes, *E* denotes the set of directed edges, and $e_{v_{i},v_{j}}$ denotes the edges from node $v_{i}$ to $v_{j}$.

**Definition** **2.** *Properties of Nodes and Edges.*

Edges in a road network can carry various attributes depending on the application. For example, common edge attributes include driving distance or driving time. In this paper, $d(v_{i},v_{j})$ denotes the distance from node $v_{i}$ to node $v_{j}$, while $v(v_{i},v_{j})$ represents the average speed from node $v_{i}$ to $v_{j}$. The driving time from node $v_{i}$ to node $v_{j}$ is calculated as follows (Equation (1)):

(1) $$tr\left(v_{i},v_{j}\right)=\frac{d(v_{i},v_{j})}{v(v_{i},v_{j})}.$$

Unlike traditional path planning, nodes in this paper also have attribute values; here, $p(v_{i})$ denotes the charging power of node $v_{i}$. If node $v_{i}$ is not a charging station, then $p(v_{i})=0$; otherwise, $p(v_{i})>0$.

**Definition** **3.** *Travel Route.*

As shown in Figure 1, the red path denotes a charging path. The route $R=\langle r_{0},r_{1},\ldots ,r_{m}\rangle$ is a connected path from the origin $r_{0}$ to the destination $r_{m}$, where $r_{i}\in V$ and i∈{0,1,…,m}.

**Definition** **4.** *Electricity Consumption.*

The electricity consumption from $r_{i}$ to $r_{j}$ is calculated as follows (Equation (2)):

(2) $$c\left(r_{i},r_{j}\right)=p_{a}\cdot tr\left(r_{i},r_{j}\right)+\mu \cdot d\left(r_{i},r_{j}\right)$$

where μ denotes the electricity consumption per kilometer and $p_{a}$ denotes the current power of the electrical appliances. This variable changes depending on whether the user switches appliances such as air conditioners and heaters on or off.

**Definition** **5.** *Charge Time and Charging Electricity.*

The difference between EV route planning and traditional path planning is that the charging time along the route must also be considered. The charging time needs to be determined by an algorithm. Let $tc(r_{i})$ denote the charging time at node $r_{i}$ in the route *R*; then, the charging electricity at node $r_{i}$ is provided by $c\left(r_{i}\right)=p\left(r_{i}\right)\cdot tc\left(r_{i}\right)$.

**Definition** **6.** *Waiting Time.*

EVs may need to wait in line when arriving at a charging node $r_{i}$. The waiting time at each node can vary. There have been many related studies on the estimation of waiting time [46]. The waiting time can be estimated by analyzing the previous data of the charging station through a probability distribution (Poisson, normal, etc.); however, in this article, obtaining the waiting time through probability distribution will cause the results of each experiment to change, as the waiting time is not certain. Therefore, we make an appointment at the charging station to determine the waiting time and charging time when the vehicle arrives at the charging station at a certain time in the future. This mechanism is described in detail in Section 3.2. The expected queue waiting time at node $r_{i}$ is denoted by $tw(r_{i})$; this is a function of the arrival time $ta(r_{i})$ and depends on several factors, including the number of charging piles at the current charging station, the number of EVs being charged, their charging times, and the arrival times of scheduled EVs. It is calculated as follows:

(3) $$\begin{array}{cc} ta(r_{i}) & =\sum_{j=1}^{i}\left(tr\left(r_{j-1},r_{j}\right)+tc\left(r_{j-1}\right)+tw\left(r_{j-1}\right)\right)\\ tw(r_{i}) & =\left\{\begin{array}{cc} 0, & tc(r_{i})=0\\ F[ta\left(r_{i}\right)], & tc(r_{i})>0 \end{array}\right. \end{array}$$

where $F[ta\left(r_{i}\right)]$ is a segmented function that considers factors such as the number of charging piles at the current charging station, the number of EVs being charged, and their respective charging times.

As a simple example, we can consider a charging station with two charging posts, both of which are initially empty. If the first EV starts charging at time 10 with a charging time of 30 units and the second EV starts charging at time 30 with a charging time of 20 units, then the waiting time function F(x), where *x* is the arrival time at the charging station, is provided by

(4) $$F\left(x\right)=\left\{\begin{array}{cc} 0, & 0\leq x<30\\ 40-x, & 30\leq x<40\\ 0, & x\geq 40. \end{array}\right.$$

When 0≤x<30, there are vacant charging piles, and no waiting is required. When 30≤x<40, the first EV is charging until time 40 and the second EV is charging until time 50. The minimum waiting time is until time 40, meaning that the waiting time is 40−x. When x≥40, the first EV has finished charging and there are vacant charging piles, meaning that no waiting is needed.

**Definition** **7.** *EV States.*

Each EV has a distinct state, which includes its initial electricity $E_{0}$, maximum electricity $E_{m}$, and reserved electricity at the destination $E_{r}$. The reserved electricity at the destination is the amount of electricity that the user wishes to have remaining upon reaching the destination.

**Definition** **8.** *EV Charging Route Planning Problem Based on User Requirements (EVCRP-UR).*

Given the road network G=〈V,E〉, attributes of each edge (including driving time and electricity consumption), charging power at each node $p(v_{i})$, initial and maximum electricity of the EV $E_{0}$ and $E_{m}$, reserved electricity at the destination $E_{r}$, starting node $r_{0}$, and destination node $r_{m}$, the goal of the proposed EV charging route planning problem (EVCRP-UR) is to determine an optimal route *R* and charging time $tc(r_{i})$ at each charging node in *R*. The objective is to ensure that the EV follows an appropriate path, charges at suitable stations, and reaches the destination while minimizing the total travel time, which includes driving time, charging time, and waiting time. The EVCRP-UR problem can be formulated as shown below.

(5) $$\begin{array}{cc} \min:\; & t\left(R\right)=\sum_{i=1}^{m}\left(tr\left(r_{i-1},r_{i}\right)+tc\left(r_{i}\right)+tw\left(r_{i}\right)\right) \end{array}$$

(6) $$\begin{array}{cc} s.t.\; & \forall i\in \left\{1,2,\ldots ,m\right\},e_{r_{i-1},r_{i}}\in E \end{array}$$

(7) $$\begin{array}{cc} \; & \forall i\in \left\{1,2,\ldots ,m\right\},\mathrm{if}\;p\left(r_{i}\right)=0,\mathrm{then}\;tc\left(r_{i}\right)=0,tw\left(r_{i}\right)=0 \end{array}$$

(8) $$\begin{array}{cc} \; & \forall j\in \left\{1,2,\ldots ,m-1\right\},E_{0}+\sum_{i=1}^{j-1}c\left(r_{i}\right)-\sum_{i=1}^{j}c\left(r_{i-1},r_{i}\right)\geq 0 \end{array}$$

(9) $$\begin{array}{cc} \; & E_{0}+\sum_{i=1}^{m-1}c\left(r_{i}\right)-\sum_{i=1}^{m}c\left(r_{i-1},r_{i}\right)\geq E_{e} \end{array}$$

(10) $$\begin{array}{cc} \; & \forall j\in \left\{1,2,\ldots ,m\right\},E_{0}+\sum_{i=1}^{j}c\left(r_{i}\right)-\sum_{i=1}^{j}c\left(r_{i-1},r_{i}\right)\leq E_{m} \end{array}$$

(11) $$\begin{array}{cc} \; & tc(r_{i})\geq 0 \end{array}$$

The meanings of the constraints (Equations (6)–(11)) are as follows:

(6) The edges traveled by the route *R* must belong to the set of edges; the route cannot take a path that does not exist in the network.
(7) At nodes without charging stations (where the charging power is 0), both the charging time and waiting time are 0.
(8) The EV must have enough electricity to reach each node in the route *R*.
(9) The EV must have sufficient reserved electricity upon reaching the destination node.
(10) The EV’s electricity cannot exceed its maximum electricity after charging at a charging station.
(11) The charging time of the EV at a charging station must be greater than or equal to 0.

### 3.2. System Architecture

**Figure 2:**
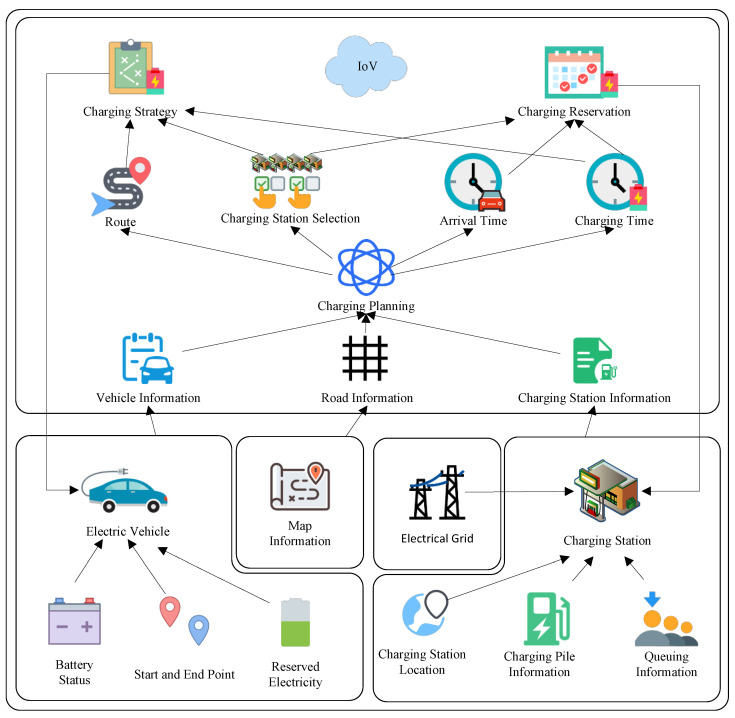
System architecture for solving the EVCRP-UR problem.

Figure 2 shows the architecture of the smart EV charging planning system, which mainly includes four modules: EV, road conditions, charging station, and Internet of Vehicles (IoV) [47]. The main contents of each module are as follows:

- **Map information:** This includes road information between origin and destination. It provides network topology diagrams electricity consumption, and time estimation on the path for charging planning.
- **EV module:** Contains the main information of EVs that currently require charging planning, including their battery status, origin and destination, and reserved electricity. The battery status mainly includes the current battery electricity, maximum battery capacity, electricity consumption rate, and other information.
- **Electric grid module:** provides electricity to charging stations and provides cost electricity price information for charging station electricity pricing.
- **Charging station module:** contains the main information of the charging stations between the origin and destination, including charging station location, charging pile information, and queuing information. Charging pile information includes the power of each charging pile, the charging price, and other information, while the queuing information includes whether there is a wait and the required waiting time.
- **IoV module:** Charging planning is based on vehicle information, road information, and charging station information. At the end of charging planning, the following information is generated: charging route, charging station selection, estimated arrival time, and charging time. A charging strategy is generated based on the charging route, charging station selection, and charging time, then the strategy is sent to the EV. If the EV agrees to the strategy, charging reservation information is generated based on the estimated arrival time and charging time, which is then sent to the selected charging station to make a reservation for the charging pile.

**Figure 3:**
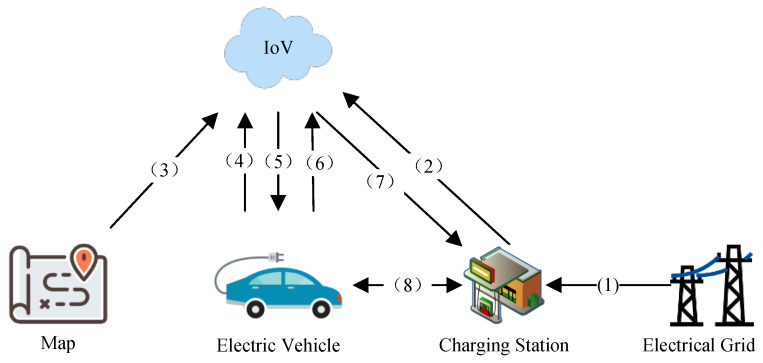
System workflow for solving the EVCRP-UR problem.

Figure 3 illustrates the system workflow of the charging route planning system for EVs. The system comprises the map and four main modules: the EV, charging station, electric grid, and Internet of Vehicles (IoV). The workflow proceeds as follows:

(1) The electric grid supplies electricity to the charging station and collects payment.
(2) The charging station updates the pricing of its charging piles based on information from the electric grid and its own status. The station also updates its own information, such as the number of available charging piles and the power of the charging piles. This updated information is then uploaded to IoV.
(3) The IoV also needs to acquire updated map information.
(4) The EV provides its own information and initiates charging planning requests.
(5) The IoV performs charging planning based on the map information, EV information, and charging pile information. The charging plan selects charging stations for the EV and determines the charging time at these stations. Upon completion, the planning results are provided to the EV.
(6) After receiving the charging planning results, the EV decides whether or not to agree to the proposed charging plan. If the vehicle agrees, it sends a charging reservation request to the IoV.
(7) The IoV forwards the charging reservation request to the charging station, completing the reservation.
(8) Upon arrival at the charging station, the EV charges at the station. The charging station collects the fee, completing the transaction.

### 3.3. Road Network Optimization

In this subsection, we introduce a road network optimization method to improve the efficiency of the EV charging route planning process.

A road network for EV routing typically consists of a large number of nodes, with each node representing a point such as an intersection or a charging station. However, a significant portion of these nodes may not participate in charging-related operations (i.e., they are non-charging nodes). Although present in the network, these non-charging nodes do not directly contribute to the charging strategy, and can create unnecessary computational overhead when path-planning algorithms process the entire network; as a result, the total number of nodes and edges increases substantially, which not only makes the path-planning algorithm more complex but also increases the computational time and memory usage. Therefore, optimizing the road network by removing non-charging nodes can improve efficiency and reliability thanks to the reduction in unnecessary data processing.

The proposed road network optimization method works by systematically removing non-charging nodes and the edges connecting them. This approach significantly reduces the overall size of the road network, which in turn lowers the computational complexity and speeds up the path planning process. By eliminating unnecessary nodes and edges, we ensure that the focus remains on critical nodes. The benefits of road network optimization are as follows:

- **Reduction in computational complexity and improved efficiency:** By removing non-charging nodes and irrelevant edges, the algorithm only needs to process those nodes and edges that directly impact the EV’s journey, such as charging stations or key road intersections. This reduces the overall number of nodes to evaluate, accelerating the path planning process. This streamlined network ensures that the routing algorithm focuses only on the most relevant paths, enhancing both the efficiency and reliability of the solution.
- **Avoiding power depletion:** A key consideration in EV path planning is to avoid running out of power during travel. By optimizing the road network, we ensure that the algorithm focuses on paths where charging stations are accessible and that unreachable areas or disconnected segments are removed from the search. This guarantees that the EV can always find feasible charging stations along its route, preventing any scenarios where the vehicle might run out of power without any charging options available.
- **Improved route selection and reliability:** By focusing only on charging-related nodes and reachable roads, the optimization method ensures that the routing algorithm selects paths that maximize travel efficiency while accounting for charging needs. The removal of irrelevant nodes and edges increases the reliability of the solution, as it reduces the risk of selecting unnecessary detours or paths that are disconnected from the charging infrastructure.

The following is the specific process of road network optimization:

#### 3.3.1. Remove Non-Charging Nodes

When an electric car needs to be charged, the main consideration is which charging node to use. To decide which path to take to reach the selected charging node, we choose the path with the smallest weight, which can be obtained through the Dijkstra algorithm.

**Figure 4:**
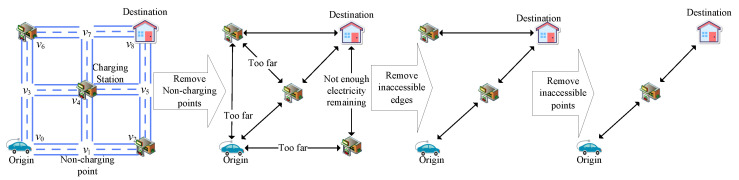
Road network optimization.

As shown in Figure 4, after finding the shortest weight between the origin, each charging station, and the destination, we remove the other paths. By removing the non-charging nodes in the middle, we obtain the edges that can be directly reached by each node. Finally, a topological graph is obtained in which the node set only has the origin, destination, while charging node, and the edge set E only has the edges with the shortest weight between them.

#### 3.3.2. Remove Inaccessible Edges

According to the initial electricity, the maximum electricity of the EV, and the electricity consumption between each node, we remove the edges that the EV cannot reach to simplify the topology and prevent the EV’s charge from running out.

As shown in Figure 4, when an EV starts from the origin, if its electricity consumption $c(r_{0},v_{i})$ from the origin $r_{0}$ to a charging station $v_{i}$ is greater than the initial electricity $E_{0}$, then the edge between the origin and that charging station $e_{r_{0},v_{i}}$ is removed from the edge set E, as shown in the following formula:

(12) $$\begin{array}{c} \forall v_{i}\in V,if\;\;c\left(r_{0},v_{i}\right)>E_{0},then\;E=E-e_{r_{0},v_{i}}. \end{array}$$

When an EV departs from any charging station $v_{i}$, if the electricity consumption $c(v_{i},v_{j})$ from charging station $v_{i}$ to any node $v_{j}$ is greater than the maximum electricity $E_{m}$, this indicates that the node in question cannot be reached even with a full charge; thus, edge $e_{v_{i},v_{j}}$ is removed from the set of edges, as follows (Equation (13)):

(13) $$\begin{array}{c} \forall v_{i},v_{j}\in V,if\;\;c\left(v_{i},v_{j}\right)>E_{m},then\;E=E-e_{v_{i},v_{j}}. \end{array}$$

As an EV travels from any charging station $v_{i}$ to its destination $r_{m}$, if the maximum possible remaining electricity $E_{m}-c\left(v_{i},r_{m}\right)$ from charging station $v_{i}$ to the destination $r_{m}$ is less than the reserved electricity $E_{r}$, this means that the EV does not have enough charge to reach the destination with the required reserved electricity even if it is fully charged. In this case, edge $e_{v_{i},r_{m}}$ is removed from the edge set, as follows (Equation (14)):

(14) $$\begin{array}{c} \forall v_{i}\in V,if\;\;E_{m}-c\left(v_{i},r_{m}\right)<E_{e},then\;E=E-e_{v_{i},r_{m}}. \end{array}$$

#### 3.3.3. Remove Inaccessible Nodes

As shown in Figure 4, after removing some edges, it may be the case that certain charging stations are unreachable or cannot be reached without passing through the destination. In these cases, we directly remove the node in question and the edges connecting to it.

### 3.4. Improved Ant Colony Optimization (IACO)

Given the complexity of EVCRP-UR, which not only involves optimizing driving routes while satisfying various constraints but also considers the selection of charging stations and charging time, this problem presents significant challenges. The most commonly used algorithms today are graph search [48], shortest path [36,49], heuristic search [38,50], and similar algorithms. However, existing algorithms only consider the path length problem, which cannot directly solve the problem presented in this paper. While small instances of the problem can be solved using exact algorithms, heuristic and metaheuristic methods are generally more suitable due to their ability to handle larger problem sizes efficiently. However, no single algorithm can solve all problems perfectly, and each approach comes with its strengths and limitations.

Ant colony optimization (ACO) is widely recognized for its effectiveness in solving graph-based pathfinding problems. However, traditional ACO struggles with the added complexities of queuing systems, charging time selection, and incorporating user preferences in the decision-making process. Dynamic programming (DP), while effective in optimizing the charging time selection, does not assist in optimizing the path itself. This highlights the need for a combined approach that leverages the strengths of different techniques to overcome these challenges.

To address these shortcomings, we propose an improved ant colony optimization (IACO) algorithm for EV charging route planning that integrates multiple techniques to enhance both the routing and charging time optimization processes. Our approach combines ACO with dynamic programming and a new topology optimization method to effectively reduce computational complexity and improve path planning efficiency. The key innovations in our methodology are as follows:

- **Comprehensive heuristic function:** We propose a novel heuristic function that incorporates three factors, namely, path deviation value, charging station importance, and destination proximity. This comprehensive approach evaluates the value of each node based on these multiple criteria, ensuring that the algorithm considers not just the path length but also the strategic location of charging stations and their relevance to the overall route.
- **Charging time optimization:** To address the challenge of efficiently selecting charging times, we propose a discrete electricity dynamic programming (DE-DP) algorithm. This algorithm calculates the optimal charging plan for each path, integrating the charging times into the overall route planning and minimizing unnecessary delays. Moreover, the algorithm can adaptively adjust the charging times based on the user’s remaining electricity requirements at the destination. By optimizing the charging time, the DE-DP algorithm helps reduce the total travel time, thereby enhancing the overall efficiency of EV route planning.
- **Improved probability weight calculation:** We enhance the traditional ACO method by adjusting the probability weight calculation to account for whether the selected node is the destination. This modification ensures that the heuristic function remains effective even when the value of the node at the destination is zero, addressing a common limitation in classical ACO implementations. This improvement guarantees that the algorithm assigns proper weight to the destination node, ensuring that paths leading to the destination are prioritized.

By combining the strengths of ant colony optimization, dynamic programming, and topology optimization, our improved ant colony optimization (IACO) algorithm overcomes the inherent limitations of individual methods. This integrated approach not only improves path selection but also optimizes charging times, addressing the challenges of the proposed EVCRP-UR problem.

The specific process of the IACO algorithm is as follows.

#### 3.4.1. Initialization

Initialize the relevant parameters, including the number of ants, pheromone factor, etc. At moment t = 0, the ants are placed at the origin of the EV and the pheromone concentration of each edge is initialized as follows (Equation (15)):

(15) $$\begin{array}{c} \tau_{v_{i},v_{j}}\left(0\right)=\tau_{0} \end{array}$$

where $\tau_{0}$ is the initial pheromone concentration from node $v_{i}$ to $v_{j}$.

#### 3.4.2. Calculate Heuristic Factor

Unlike the basic ant colony algorithm, which uses $\eta_{v_{i},v_{j}}=1/d_{v_{i},v_{j}}$ to denote the heuristic factor, we need to consider the location of the charging station and its power, along with many other elements. First, we want the ants to choose charging stations that do not deviate in the direction of travel. We use $dev(v_{i},v_{j})$ to denote the path deviation value from node $v_{i}$ through $v_{j}$ to the destination, as follows (Equation (16)):

(16) $$\begin{array}{c} dev\left(v_{i},v_{j}\right)=1/\left(tr\left(v_{i},v_{j}\right)+tr\left(v_{j},v_{e}\right)-tr\left(v_{i},v_{e}\right)+1\right) \end{array}$$

where the range of $dev(v_{i},v_{j})$ is (0, 1]. This value indicates the extent to which we have to spend more path time if $v_{j}$ is chosen as the next charging node from the current node. A larger value indicates a smaller amount of extra time spent on the elapsed driving time. A value of 1 indicates that $v_{j}$ is exactly on the shortest path from the current node to the destination, and will not take more time.

Because we also want the ants to move as close as possible to the destination, we use $d_{end}\left(v_{i},v_{j}\right)$ to denote the degree of proximity to destination, which is the ratio of the distance from the node $v_{i}$ to destination minus the distance from the node $v_{j}$ to destination to the distance from the node $v_{i}$ to destination. The range of values is [−∞, 1]. We map it to the interval (0, 1], which we denote as $d_{end}$ (Equation (17)):

(17) $$\begin{array}{cc} d_{end}^{\prime }\left(v_{i},v_{j}\right) & =\left(tr\left(v_{i},v_{e}\right)-tr\left(v_{j},v_{e}\right)\right)/tr\left(v_{i},v_{e}\right)\\ d_{end}\left(v_{i},v_{j}\right) & =exp^{d_{end}^{\prime }\left(v_{i},v_{j}\right)-1}. \end{array}$$

With the above information and the power information of the charging station, we can obtain the heuristic factor function as follows (Equation (18)):

(18) $$\begin{array}{cc} \; & \eta_{v_{i},v_{j}}\left(t\right)=\left\{\begin{array}{cc} {\left[dev\left(v_{i},v_{j}\right)\right]}^{a}\cdot {\left[p\left(v_{j}\right)\right]}^{b}\cdot {\left[d_{end}\left(v_{i},v_{j}\right)\right]}^{c} & ,v_{j}\neq v_{e}\\ \eta_{v_{i},J\left(v_{i}\right)-v_{e}}^{m}\left(t\right) & ,v_{j}=v_{e} \end{array}\right. \end{array}$$

where $p(v_{j})$ denotes the charging power of $v_{j}$. The probability of a node being selected is considered based on $d_{end}\left(v_{i},v_{j}\right)$, $dev(v_{i},v_{j})$, and the charging power of $v_{j}$. The goal is to make the ants choose a point that does not deviate from the shortest path, has the highest charging power possible, and is as close to the destination as possible. Here, a,b,c denote the path importance factor, charging station importance factor, and destination proximity importance factor, respectively, $J(v_{i})$ denotes the set of nodes that can be reached from node $v_{i}$, and $\eta_{v_{i},J\left(v_{i}\right)-v_{e}}^{m}\left(t\right)$ denotes the maximum value of the heuristic factor from point $v_{i}$ to all points in $J(v_{i})$ (except the end point) at time t. In addition to choosing the charging station, ants can also choose to travel directly to the destination when it is reachable.

#### 3.4.3. Choose Path

Each ant determines which edge to choose based on the heuristic factor and pheromone concentration. The next node is chosen according to the pseudorandom proportional rule. In this way, ants are more likely to select states with higher pheromone concentration and that are more favorable for solving the problem, but also retain a certain degree of randomness to ensure diversity in the search. Starting from $v_{i}$, the probability weight of the k-th ant choosing the next node as j at moment t is as follows (Equation (19)):

(19) $$\begin{array}{cc} \; & W_{v_{i},v_{j}}^{k}\left(t\right)=\left\{\begin{array}{c} {\left[\tau_{v_{i},v_{a}}\left(t\right)\right]}^{\alpha }{\left[\eta_{v_{i},v_{a}}\left(t\right)\right]}^{\beta },v_{j}\in J_{k}\left(v_{i}\right)-v_{e}\\ exp^{max(m-n,1)}{\left[\tau_{v_{i},v_{a}}\left(t\right)\right]}^{\alpha }{\left[\eta_{v_{i},v_{a}}\left(t\right)\right]}^{\beta },v_{j}=v_{e}\\ 0,orthers \end{array}\right. \end{array}$$

where $J_{k}\left(v_{i}\right)$ denotes the set of nodes that ant k has not yet traveled to and can reach from $v_{i}$, ‘reachable nodes’ means those nodes that the ant can travel to after being fully charged at node $v_{i}$, $\tau_{v_{i},v_{a}}$ is the pheromone concentration from node $v_{i}$ to $v_{j}$, $\eta_{v_{i},v_{a}}$ is the heuristic factor from node $v_{i}$ to $v_{j}$, the parameters α, β respectively denote the pheromone importance factor and heuristic importance factor, m is the number of charging stations currently charged, and n is the desired number of charging times for the EV. The probability weight of the destination being selected increases when the desired number of charges is exceeded.

If the random number q≤ the pseudorandom factor $q_{0}$, then the ant directly selects the next node that maximizes the probability weight, as follows (Equation (20)):

(20) $$\begin{array}{c} v_{j}=\underset{v_{a}\in J_{k(v_{i})}}{argmax}\left(W_{v_{i},v_{j}}^{k}\left(t\right)\right). \end{array}$$

If the random number $q>q_{0}$, then the next node is chosen according to the probability $P_{v_{i},v_{j}}^{k}$ of ant k moving from node $v_{i}$ to $v_{J}$, as shown below (Equation (21)).

(21) $$\begin{array}{c} P_{v_{i},v_{j}}^{k}\left(t\right)=\left\{\begin{array}{cc} W_{v_{i},v_{j}}^{k}\left(t\right)/\sum_{v_{a}\in J_{k}\left(v_{i}\right)}W_{v_{i},v_{j}}^{k}\left(t\right), & v_{j}\in J_{k}\left(v_{i}\right)\\ 0, & others \end{array}\right. \end{array}$$

#### 3.4.4. Calculate Charging Time and Total Time

Using the above method, each ant can obtain the path to reach the destination from the origin. Next, we choose the appropriate charging time for each ant’s path.

The original problem with route R is uncertain. The charging time $tc(r_{i})$ is also uncertain, as we do not know which path will take less time to charge, and R contains non-charging nodes. After the ant reaches the end, a deterministic path is obtained.

For any determined route R, the problem is transformed into a nonlinear programming problem that satisfies the constraints. Among them, $tc(r_{i})$ is an unknown number. The constraints and objective function are as follows.

(22) $$\begin{array}{cc} min: & t\left(R\right)=\sum_{i=1}^{m}\left(tc\left(r_{i}\right)+tw\left(r_{i}\right)\right) \end{array}$$

(23) $$\begin{array}{cc} s.t. & \forall j\in \left\{1,2,\cdots ,m-1\right\},E_{0}+\sum_{i=1}^{j-1}c\left(r_{i}\right)-\sum_{i=1}^{j}c\left(r_{i-1},r_{i}\right)\geq 0 \end{array}$$

(24) $$\begin{array}{cc} \; & E_{0}+\sum_{i=1}^{m-1}c\left(r_{i}\right)-\sum_{i=1}^{m}c\left(r_{i-1},r_{i}\right)\geq E_{e} \end{array}$$

(25) $$\begin{array}{cc} \; & \forall j\in \left\{1,2,\cdots ,m\right\},E_{0}+\sum_{i=1}^{j}c\left(r_{i}\right)-\sum_{i=1}^{j}c\left(r_{i-1},r_{i}\right)\leq E_{m} \end{array}$$

(26) $$\begin{array}{cc} \; & tc(r_{i})\geq 0 \end{array}$$

We design a discrete electricity dynamic programming (DE-DP) algorithm to solve this problem. We acknowledge that the scalability of dynamic programming (DP) can be a limitation, particularly in problems with increasing variables such as the number of charging stations, nodes, edges, and cars. To address this, our DE-DP approach incorporates preprocessing steps such as removing inaccessible nodes and edges in order to reduce the problem size. Furthermore, the state space is discretized to ensure manageable computational complexity. However, we recognize that as the problem size grows, the computational demands of DP still could become prohibitive. To mitigate this, we explore hybrid methods that combine DP with IACO in order to efficiently handle large-scale scenarios. The number of charging stations on the path obtained by the IACO algorithm is not actually large, as the IACO algorithm selects better charging stations through heuristic functions and pheromones. Therefore, the actual problem size of the DP algorithm after the above methods is not large. The number of charging stations in the path obtained by the IACO algorithm is basically less than 20, and even less when the algorithm converges at the end. The algorithm is described below.

Given the origin $r_{0}$, initial electricity $E_{0}$, destination $r_{m}$, and reserved electricity at the destination $E_{r}$, the total electricity required to travel from the origin to the destination is

(27) $$C_{n}=Max\left(c\left(r_{0},r_{m}\right)+E_{e}-E_{0},0\right).$$

We divide the total electricity that needs to be charged into *L* parts for charging. The electricity size of each part is

(28) $$C_{L}=C_{n}/L.$$

**(1) State definition:** Using F[i][j](i=0,1,…,n;j=0,1,2,…,L) to denote the shortest charging time and waiting time for stopping at only the first i charging stations to charge $j\cdot C_{L}$ amounts of electricity, and using tc[i][j][c] and tw[i][j][c] to denote the charging and waiting times at the c-th charging station when F[i][j] is optimal, our goal is to find the optimal shortest charging time and waiting time $F\left[n\right]\left[C_{L}\right]$ for stopping at the first n charging stations to charge an amount of electricity $L\cdot C_{L}$, where n is the number of charging stations (n = m − 1).

**(2) Initialization:** We initialize F[i][j] to be all positive infinity, and initialize tc[i][j] and tw[i][j] to be all 0. When j=0, the shortest charging time and waiting time for any i to charge 0 copies of electricity are both 0 (i.e., f[i][0]=0).

**(3) State transfer:** For any i>0,j≥0 situation, we choose to charge $k\cdot C_{L}$ amount of electricity at the i-th charging station. The remaining electricity arriving at the i-th charging station is determined by (Equation (29)):

(29) $$c_{i}=E_{0}+\left(j-k\right)*C_{L}-c\left(r_{0},r_{i}\right).$$

The maximum charging electricity at the *i*-th charging station is determined by (Equation (30)).

(30) $$ch_{i}^{m}=E_{m}-c_{i}.$$

State transfer must meet the following four conditions:

(1) The remaining electricity arriving at the *i*-th charging station is less than or equal to the possible maximum remaining electricity (Equation (31)):
  (31) $$c_{i}\leq E_{m}-c\left(r_{i-1},r_{i}\right).$$
(2) The *i*-th charging station $r_{i}$ can be reached (Equation (32)):
  (32) $$c_{i}\geq 0.$$
(3) The charging electricity $ch_{i}^{m}$ is less than or equal to the maximum electricity demand (Equation (33)):
  (33) $$ch_{i}^{m}\leq k\cdot C_{L}.$$
(4) There is a solution for charging $\left(j-k\right)\cdot C_{L}$ amount of electricity at the first *i*−1 charging stations (Equation (34)):
  (34) f[i−1][j−k]≠+∞.

If the conditions are met, then the time to arrive at the *i*-th charging station is determined by (Equation (35)):

(35) $$ta_{i}=tr\left(r_{0},r_{i}\right)+f\left[i-1\right]\left[j-k\right].$$

The charging time at the *i*-th charging station is determined by (Equation (36)):

(36) $$cht_{i}=k\cdot C_{L}/p\left(r_{i}\right).$$

The charging waiting time at the *i*-th charging station is determined by (Equation (37)), with $tws(i,ta_{i})$ denoting the charging waiting time required to arrive at the *i*-th charging station at time $ta_{i}$:

(37) $$tw_{i}=tws\left(i,ta_{i}\right).$$

Then, when the *i*-th charging station charges $k\cdot C_{L}$ amount of electricity, the total charging time and waiting time is *t* (Equation(38)):

(38) $$t=f\left[i-1\right]\left[j-k\right]+cht_{i}+tw_{i}.$$

Then, the state transition equation is as follows (Equation(39)):

(39) f[i][j]=min(f[i−1][j],t).

In addition, tc[i][j] and tw[i][j] are updated along with the state transition equation; see Algorithm 1 for details. We finally obtain the optimal charging time $tc(r_{i})$ at each charging station under route R.
**Algorithm 1:** Discrete Electricity Dynamic Programming (DE-DP)
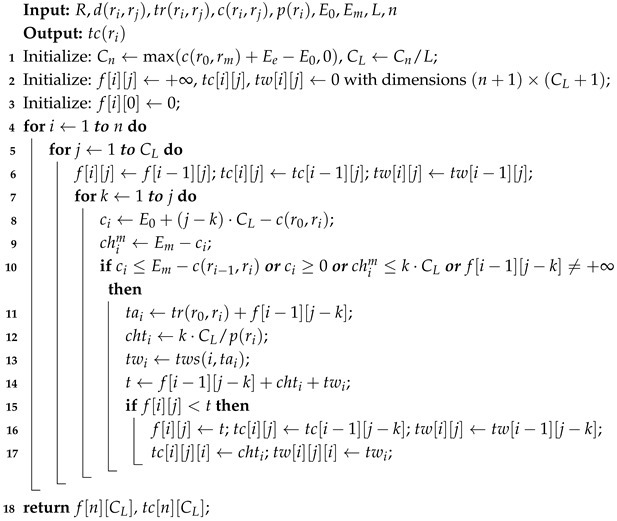


#### 3.4.5. Pheromone Update

To better explore the problem space, we use pheromone volatilization to prevent the ants from relying too much on earlier paths. This can help the ant colony algorithm to find the globally optimal solution. Pheromone release allows the ants to communicate with each other. The latter ants then move along paths with higher pheromone concentrations. After all ants reach their destinations, we update the pheromone of the path they passed through. Ant k locally updates the pheromone concentration after reaching the destination according to the following rule (Equation (40)):

(40) $$\begin{array}{cc} \; & \tau_{v_{i},v_{j}}\left(t+1\right)=\left(1-\xi \right)\cdot \tau_{v_{i},v_{j}}\left(t\right)+\xi \cdot \Delta \tau_{v_{i},v_{j}}^{k}\\ \; & \Delta \tau_{v_{i},v_{j}}^{k}=\left\{\begin{array}{cc} Q/T, & Ant\;k\;passes\;through\;edge\;e_{v_{i},v_{j}}\\ 0, & others \end{array}\right. \end{array}$$

where ξ is the volatilization factor of the local pheromone, $\Delta \tau_{v_{i},v_{j}}^{k}$ is the incremental amount of pheromone released by ant k on the edge $e_{v_{i},v_{j}}$, *Q* is the pheromone constant, and *T* denotes the total time spent by this ant to reach its destination.

When the entire ant colony completes a search, the pheromone on the globally optimal path is updated as follows (Equation (41)):

(41) $$\begin{array}{cc} \; & \tau_{v_{i},v_{j}}\left(t+1\right)=\left(1-\rho \right)\cdot \tau_{v_{i},v_{j}}\left(t\right)+\rho \cdot \Delta \tau_{v_{i},v_{j}}\\ \; & \Delta \tau_{v_{i},v_{j}}=\left\{\begin{array}{cc} Q/T_{best}, & e_{v_{i},v_{j}}\;belongs\;to\;optimal\;path\\ 0, & others \end{array}\right. \end{array}$$

where ρ is the global pheromone evaporation rate, $\Delta \tau_{v_{i},v_{j}}$ is the pheromone increment released by the globally optimal path on edge $e_{v_{i},v_{j}}$, and $T_{best}$ denotes the time to reach a destination through the globally optimal path.

The pseudocode of the ant colony algorithm is provided in the following Algorithm 2.
**Algorithm 2:** Improved Ant Colony Optimization (IACO)
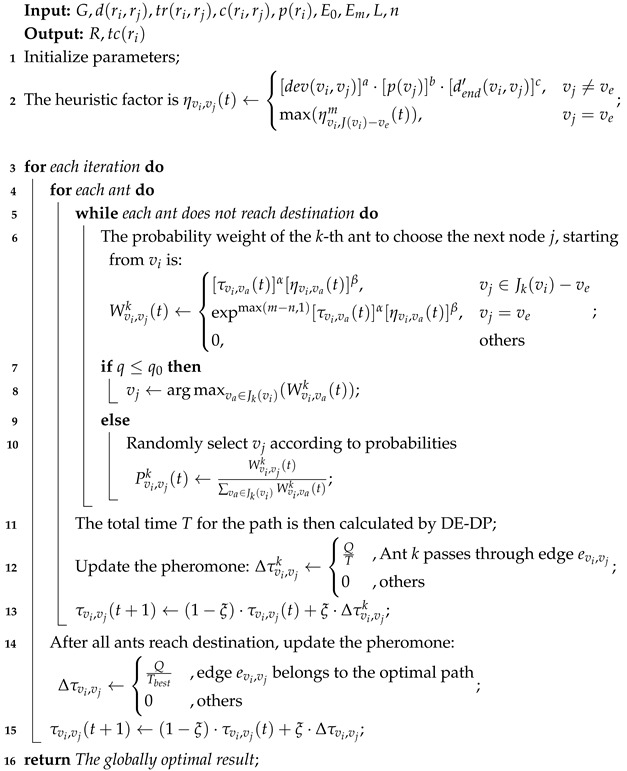


## 4. Dataset

### 4.1. Beijing Road Network Dataset

**Figure 5:**
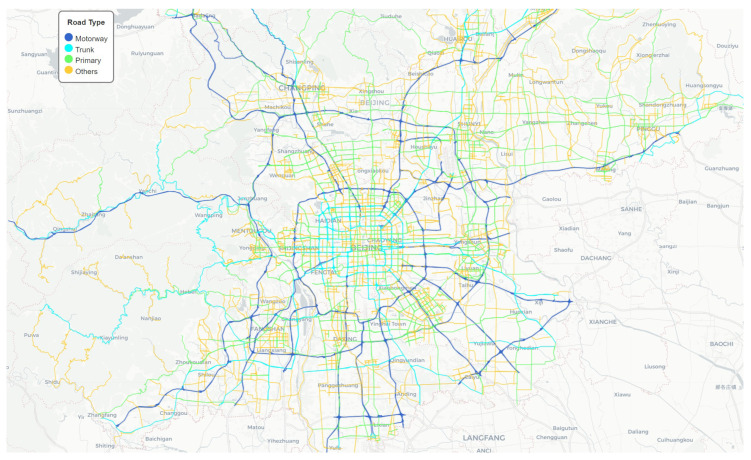
Beijing Road network.

We collected road network data of Beijing, China from OpenStreetMap (https://www.openstreetmap.org/ (accessed on 3 September 2024)) [51]. We obtained the data through OSMnx [52]. The data consisted of 15,170 nodes and 25,387 edges, including information such as the starting and ending points of roads, road types, and road lengths. The road types mainly include the motorway, trunk, primary, and other types, as shown in Figure 5.

### 4.2. EV Public Charging Stations Dataset

**Figure 6:**
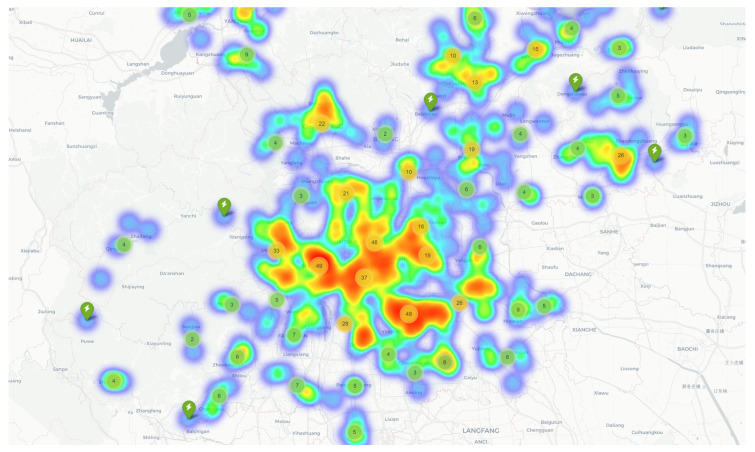
Distribution of public charging stations in Beijing.

We collected charging station data for Beijing from ChargeBar [32], resulting in 2072 charging stations. Each EV charging station’s information contains the charging station address, latitude and longitude coordinates of the charging station, and number of fast charging piles and slow charging piles. We used the 653 public charging stations as our dataset, as shown in Figure 6.

### 4.3. EV Data

The EV data include the origin and destination, maximum electricity, initial electricity, reserved electricity at the destination, power consumption of electrical appliances, electricity consumption per kilometer, and straight-line distance between the origin and destination. Due to the variety of EVs and the potential differences in user requirements, the data for EVs was randomly generated according to Table 2. The parameter values are provided in the form of intervals, indicating that they were randomly generated within these ranges. The parameters for the simulation were generated using uniform random sampling within predefined ranges. These ranges were chosen based on typical values observed in real-world electric vehicle information. The use of uniform sampling ensures an unbiased distribution of values within the specified ranges, allowing the simulation to encompass diverse but realistic scenarios. These parameter ranges were selected to mimic typical conditions faced by electric vehicles in various contexts. While we acknowledge that electricity consumption is variable depending on the geography of the road (hills, elevation, vehicle speed, highway driving, etc.), the electricity consumption per kilometer (µ) in the current experiment varies only due to differences between electric vehicles. This is because our road network dataset does not contain this detailed information, making it impossible to determine the actual electricity consumption of each route. Ways to calculate the actual electricity consumption of different cars based on the geographical location of the road is a problem worthy of further study. Future work could extend this model by integrating variable energy consumption and leveraging real-time geographic and traffic data to enhance both accuracy and practical relevance.

## 5. Results

### 5.1. Algorithm Parameters

To evaluate the impact of the discretization parameter *L* on the performance of DE-DP, we conducted a series of experiments. The parameter *L* determines the granularity of the discretization of the state space, which directly affects both the computational complexity and the accuracy of the solution. A finer discretization (larger *L*) can yield more precise results at the cost of increased computational time, whereas a coarser discretization (smaller *L*) reduces computational time but may compromise solution quality. This experiment aims to analyze the tradeoff between computational efficiency and solution accuracy, providing insights into the optimal choice of *L* for practical applications.

Because the number of charging stations in the path obtained by the IACO algorithm is basically less than 20, and even less when the algorithm converges at the end, we chose to conduct experiments when the number of charging stations on the path was 20. Thus, we selected ten different paths for the experiment, analyzed the results of different *L* values, and finally chose a value with better time and effect, ensuring that the IACO algorithm could achieve better results in subsequent experiments.

**Figure 7:**
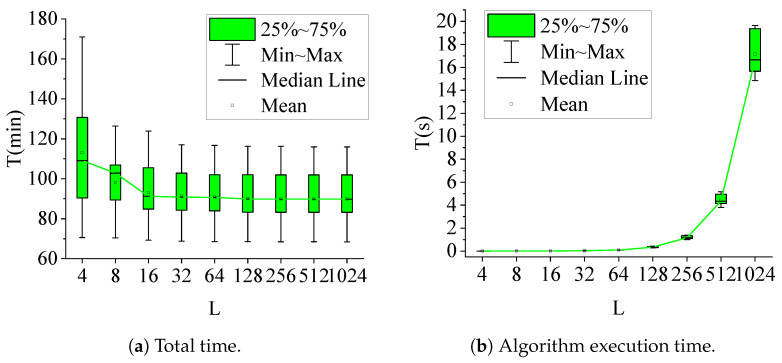
Total time and DE-DP algorithm execution time under different parameters *L*.

In Figure 7, The abscissa is the value of *L* and the ordinate is the total time and algorithm running time of the electric vehicle from the startpoint to the endpoint. Figure 7a shows that the total time calculated by the algorithm decreases as the *L* value increases, with the decrease becoming smaller and smaller; when L>64, the decrease is very small. Figure 7b shows that as the *L* value increases, the algorithm running time increases; when L>64, the algorithm running time increases significantly. Therefore, we chose L=64 as the *L* parameter value of DE-DP.

Various parameters in IACO can have a great impact on the experimental results, so it is crucial to choose good parameters. When the optimal solution does not change in 10 iterations, we consider the algorithm converged and stop the execution. Optuna [53] is a Python library for hyper-parameter optimization, which helps us to automate the selection of optimal hyper-parameter combinations. In this paper, we use Optuna to tune the ant colony algorithm, and the parameters of IACO algorithm obtained are shown in Table 3.

To evaluate the impact of different parameters on the algorithm, we conducted experiments on the effects of parameters *a* and *b* under various values. Except for the compared parameters, all other parameters were set to the values listed in Table 3.

**Figure 8:**
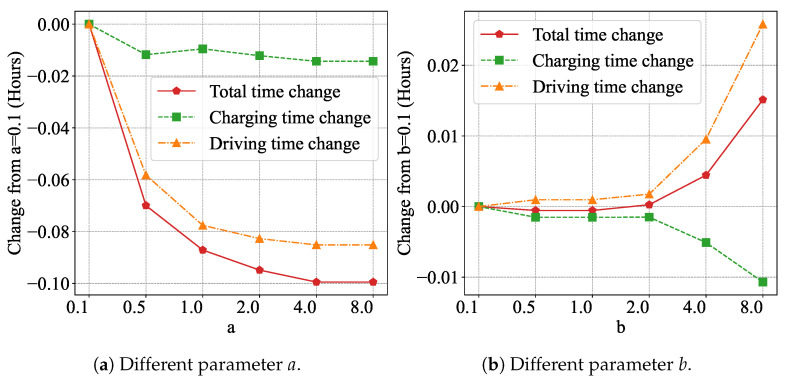
Total, charging, and driving times under different parameter values.

In Figure 8a, the *x*-axis represents the values of the parameters, while the *y*-axis shows the change in results compared to when the parameter is set to 0.1. It can be observed that increasing parameter *a* significantly reduces the driving time and total time, and slightly reduces the charging time. This is because a higher *a* increases the likelihood of selecting a better path. A better path not only reduces driving time but also lowers energy consumption, which in turn decreases the amount of energy required for charging.

As shown in Figure 8b, increasing the value of parameter *b* can reduce charging time by focusing more on fast charging. However, this emphasis on fast charging may lead to suboptimal paths, resulting in increased driving time. When *b* exceeds 2, the increase in driving time and decrease in charging time become very obvious. However, the increase in driving time is faster than the decrease in charging time, resulting in an overall increase in total time.

### 5.2. Effectiveness Evaluation

In this subsection, we study the effectiveness of our proposed IACO method by comparing it with four other baselines.

Below, we introduce the four baseline algorithms used for comparison. By comparing these baselines with the proposed IACO algorithm, we can better evaluate the performance improvements that it offers.

- **Baseline 1: Nearest charging station (NCS) charging [35]**. When charging is required, the nearest charging station is chosen. If a vehicle simply chooses the nearest charging station, it may encounter situations in which there are too many vehicles at the charging station, resulting in long waiting times for service. EVs may also encounter situations where the charging pile power is not high.
- **Baseline 2: Improved Dijkstra algorithm (IDA) [42]**. Unlike the original Dijkstra algorithm, the IDA is specifically optimized for EV charging problems by considering factors such as charging speed at stations, remaining battery level, and charging station distribution. When the remaining battery of the electric vehicle is insufficient to reach the destination, the system calculates the routes to various charging stations, considering the waiting time, charging time, and driving time, and selects the charging station with the shortest overall time. After charging, the vehicle continues to plan its route and eventually reaches the destination.
- **Baseline 3: Low battery threshold charging (LBTC)**. The vehicle prompts charging when the battery level drops below a certain percentage. Similar to IDA, this system helps users to select the charging station with the shortest total time.
- **Baseline 4: Earliest completion charging (EFC) algorithm [35,54]**. This algorithm consider the average velocity of the traffic network, the queuing situation of the charging stations, and the distance between the EV and a charging station. The charging station that can finish the charging service at the earliest time is selected.

Table 4 and Table 5 show the total time results of the three algorithms under different distances and different amounts of reserved electricity $E_{r}$, including average time, maximum time, and minimum time. Three of the algorithms have almost no difference in minimum time, but a large difference in maximum time. Table 6 presents the total time results of all five algorithms. The results are summarized as follows:

- **Minimum time:** All algorithms exhibit similar minimum total times, ranging between 1.45 and 1.52 h. This indicates that all methods perform comparably under ideal conditions, such as for short distances or minimal charging requirements.
- **Maximum time:** The maximum total time shows significant variation among the algorithms. IACO achieves the lowest maximum time of 5.12 h, followed by IDA (7.05 h), LBTC (7.26 h), EFC (8.13 h), and NCS (8.58 h). This demonstrates that IACO is more effective at handling scenarios requiring multiple charges or involving heavy traffic, whereas other algorithms may struggle with suboptimal station selection or long queues.
- **Average time:** IACO achieves the shortest average time of 3.24 h, demonstrating an 11.1% reduction compared to IDA (3.6 h) and a 20.0% reduction compared to LBTC (3.92 h). EFC and NCS have longer average times of 4.14 and 4.31 h, respectively, indicating less efficient overall performance.

**Figure 9:**
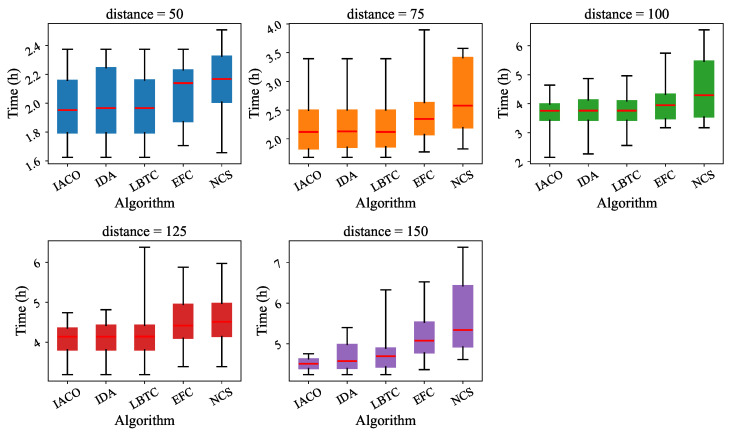
Comparison of total time results of the different algorithms at different distances.

**Figure 10:**
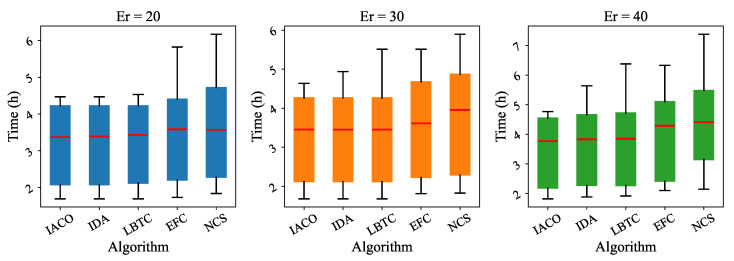
Comparison of total time results of the different algorithms at different $E_{r}$.

As shown in Figure 9 and Figure 10, the box plots for different algorithms under varying distances and reserved electricity ($E_{r}$) highlight the advantages of IACO. First, IACO consistently exhibits lower median total times than the other algorithms, regardless of the distance or $E_{r}$. Second, the interquartile range (IQR) of IACO is narrower, indicating more stable performance with less variability across different scenarios. This contrasts with the other algorithms, which show wider IQRs and higher outliers, especially under longer distances or larger $E_{r}$, reflecting their susceptibility to suboptimal decisions. Lastly, IACO effectively minimizes extreme values (outliers), demonstrating its robustness and reliability in handling complex conditions such as high traffic, multiple charges, or long distances, where the other algorithms struggle. These features make IACO a superior choice for electric vehicle charging path optimization.

**Figure 11:**
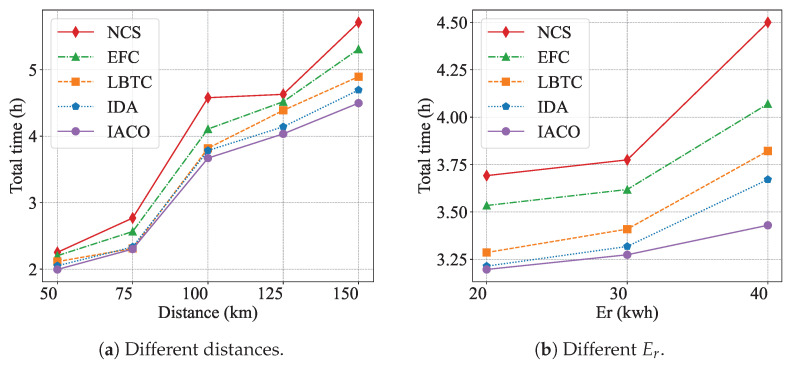
Total time results of the different algorithms at different distances and different reserved electricity to destination $E_{r}$.

As shown in Figure 11, the results indicate that as the travel distance and reserved electricity $E_{r}$ increase, the performance gap between IACO and the other algorithms becomes more pronounced. In Figure 11a, IACO maintains a consistently lower total time than other methods, with the difference growing significantly at longer distances (e.g., 125 km and 150 km). Similarly, in Figure 11b, the total time for the EFC, NCS, IDA, and LBTC algorithms rises more sharply as $E_{r}$ increases than for IACO, particularly at $E_{r}=40$, where IACO demonstrates a much more significant advantage. This highlights IACO’s superior global optimization capability, which allows it to effectively handle more complex scenarios.

## 6. Conclusions

This paper proposes the electric vehicle charging route planning based on user requirements (EVCRP-UR) problem, which comprehensively considers user needs and multiple constraints to provide personalized and practical solutions. The problem model introduces innovations such as appointment-based queuing to improve the accuracy of queuing time estimation, integration of energy consumption from in-car appliances, and user-defined reserved electricity requirements at the destination. These enhancements ensure that the proposed model aligns more with real-world application scenarios, improving user experience and satisfaction.

To address the complexities of the proposed EVCRP-UR problem we develop an improved ant colony optimization (IACO) algorithm that integrates topology optimization, a discrete electricity dynamic programming (DE-DP) algorithm for charging time planning, and enhanced heuristic factors. This hybrid approach effectively balances the strengths and weaknesses of different optimization methods, resulting in more efficient and accurate solutions.

We validated the proposed method on real-world road network and charging station data from Beijing, China. Our experimental results demonstrate that the proposed IACO algorithm outperforms baseline methods, reducing total travel time while meeting user-specific constraints. Furthermore, our approach significantly enhances the user experience by providing more reliable and flexible charging route plans.

In summary, this study provides a comprehensive framework and innovative algorithmic solutions for EV charging route planning, contributing to both the theoretical foundation and practical application of personalized EV path optimization. Future work may explore further refinements in dynamic traffic modeling and real-time optimization to extend the applicability of the proposed methods.

## Figures and Tables

**Table 1:** Notation.

Symbol	Definition
*G*	Road network
*V*	Set of nodes in the road network
*E*	Set of edges in a road network
$v_{i}$	The i-th node in *V*
$e_{v_{i},v_{j}}$	The edge from node $v_{i}$ to $v_{j}$
*N*	Number of nodes in *V*
$tr(v_{i},v_{j})$	Shortest path time from node $v_{i}$ to $v_{j}$
$p(v_{i})$	Charging power at the node $v_{i}$
$v(v_{i},v_{j})$	Average driving speed from node $v_{i}$ to $v_{j}$
μ	Electricity consumption per kilometer
$d(v_{i},v_{j})$	Distance from node $v_{i}$ to $v_{j}$
$c(v_{i},v_{j})$	Electricity consumption from node $v_{i}$ to $v_{j}$
*R*	EV travel route sequence
$r_{i}$	The i-th node in *R*
*m*	Number of nodes in *R* (starting from 0)
$p_{a}$	Current power of electrical appliances
$tc(r_{i})$	Charging time at node $r_{i}$
$c(r_{i})$	Charging electricity at node $r_{i}$
$tw(r_{i})$	Waiting time at node $r_{i}$
$ta(r_{i})$	Arrival time at node $r_{i}$
t(R)	Total time of the journey *R*
$E_{0}$	Initial electricity of EV
$E_{m}$	Maximum electricity of EV
$E_{r}$	Reserved electricity at destination

**Table 2:** EV data.

Parameter	Value
Maximum electricity: $E_{m}$ (kwh)	[50, 70]
Initial electricity: $E_{0}$ (kwh)	[10, 30]
Reserved electricity to destination: $E_{r}$ (kwh)	[20, 40]
Current Power of electrical appliances: $p_{a}$ (kw)	[0.5, 2]
Electricity consumption per km: μ (kwh/km)	[0.1, 0.2]
Distance between orgin and destination (km)	[50, 150]

**Table 3:** IACO parameters.

Parameter	Value
Number of Ants	50
Pheromone Importance Factor (α)	0.4
Heuristic Importance Factor (β)	3.3
Path Importance Factor (*a*)	4.5
Charging Station Importance Factor (*b*)	1.0
Destination Proximity Importance Factor *c*	0.3
Global Pheromone Evaporation Factor (ρ)	0.4
Local Pheromone Evaporation Factor (ξ)	0.2
Pseudorandom Factor ($q_{0}$)	0.9
Pheromone Deposit Constant (*Q*)	100

**Table 4:** Total time results of the different algorithms at different distances.

Distance (km)	Time (h)	IDA	LBTC	EFC	NCS	IACO
50	Average	2.05	2.11	2.2	2.25	2
	Maximun	3.18	4.33	4.33	4.46	2.59
	Minimum	1.58	1.58	1.58	1.58	1.58
75	Average	2.33	2.31	2.57	2.77	2.3
	Maximun	4.36	3.67	5.44	5	3.77
	Minimum	1.45	1.46	1.52	1.52	1.45
100	Average	3.78	3.82	4.11	4.58	3.67
	Maximun	5.75	6.61	8.13	8.58	5.12
	Minimum	1.98	1.98	1.99	1.99	1.98
125	Average	4.14	4.39	4.52	4.63	4.04
	Maximun	7.05	7.26	6.34	6.74	4.84
	Minimum	3.04	3.04	3.24	3.24	3.04
150	Average	4.69	4.9	5.31	5.71	4.5
	Maximun	5.64	6.48	7.38	8.35	4.78
	Minimum	4.19	4.19	4.31	4.31	4.19

**Table 5:** Total time results of the different algorithms at different $E_{r}$.

$E_{r}$ (kwh)	Time (h)	IDA	LBTC	EFC	NCS	IACO
20	Average	3.21	3.29	3.53	3.69	3.2
	Maximun	5.16	6.61	6.53	6.8	4.72
	Minimum	1.45	1.46	1.58	1.58	1.45
30	Average	3.32	3.41	3.62	3.77	3.27
	Maximun	5.29	6.18	6.53	6.53	4.78
	Minimum	1.52	1.52	1.52	1.52	1.52
40	Average	3.67	3.82	4.07	4.5	3.43
	Maximun	7.05	7.26	8.13	8.58	5.12
	Minimum	1.7	1.7	1.78	1.78	1.69

**Table 6:** Total time results of the different algorithms.

Time (h)	IDA	LBTC	EFC	NCS	IACO
Average	3.6	3.92	4.14	4.31	3.24
Maximun	7.05	7.26	8.13	8.58	5.12
Minimum	1.45	1.46	1.52	1.52	1.45

## Data Availability

Data are contained within the article.
